# Simvastatin Effects on Inflammation and Platelet Activation Markers in Hypercholesterolemia

**DOI:** 10.1155/2018/6508709

**Published:** 2018-10-01

**Authors:** Cristina Barale, Chiara Frascaroli, Rouslan Senkeev, Franco Cavalot, Isabella Russo

**Affiliations:** ^1^Department of Clinical and Biological Sciences, University of Turin, Turin, Italy; ^2^Metabolic Diseases and Diabetes Unit, San Luigi Gonzaga Hospital, Orbassano, Turin, Italy

## Abstract

**Background:**

Beside the lipid-lowering effect, statins slow the progression of atherosclerosis by exerting anti-inflammatory and platelet inhibiting effects. We investigated whether platelet inhibition by simvastatin correlates with the statin effects on lipid lowering, inflammation, oxidative stress, and endothelial and platelet activation.

**Methods:**

In hypercholesterolemic patients allocated to diet (n=20) or a 2-month treatment with diet plus 40 mg simvastatin (n=25), we evaluated platelet aggregating responses to ADP, collagen, and arachidonic acid (AA), the effect of aspirin on AA-induced aggregation, pro- and anti-inflammatory and atherogenic mediators (IL-1*β*, -5, -6, -7, -8, -9, -10, -12, and -13, IFN-*γ*, IP-10, Eotaxin, and sRAGE), markers of endothelium (sE-selectin, VEGF, and MCP-1) and platelet activation (sP-selectin, sCD-40L, RANTES, and PDGF-bb), and oxidative stress (8-OH-2'-deoxyguanosine).

**Results:**

After treatment, beside the improvement of lipid profile, we observed the following: a reduction of platelet aggregation to ADP (p=0.0001), collagen (p=0.0001), AA (p=0.003); an increased antiaggregating effect of aspirin in the presence of AA (p=0.0001); a reduction of circulating levels of IL-6 (p=0.0034), IL-13 (p<0.0001), IFN-*γ* (p<0.0001), VEGF (p<0.0001), sE-selectin (p<0.0001), sCD-40L (p<0.0001), sP-selectin (p=0.003), and 8-OH-2'-deoxyguanosine (p<0.0001); an increase of IL-10 and sRAGEs (p=0.0001 for both). LDL-cholesterol levels (i) positively correlated with IL-6, IFN-*γ*, E-selectin, sCD-40L, 8-OH-2'-deoxyguanosine, platelet aggregation to ADP, collagen, AA, and aspirin IC-50 and (ii) negatively correlated with IL-10 and sRAGE. In multiple regression analyses, LDL-cholesterol was the strongest predictor for most parameters of platelet reactivity.

**Conclusion:**

In primary hypercholesterolemia, simvastatin treatment reduced platelet activation and subclinical inflammation and improved endothelial dysfunction. LDL-cholesterol levels were the major correlate of platelet reactivity; however, other effects of statins may contribute to reducing the progression of atherosclerosis.

## 1. Introduction

Inhibitors of 3-hydroxy-3-methylglutaryl coenzyme A (HMG-CoA) reductase (statins) are the most relevant drugs used to lower serum cholesterol levels. In chronic therapy, they are highly effective in the prevention of cardiovascular events [1]. Furthermore, they slow the progression of atherosclerosis by mechanisms related not only to the cholesterol lowering effect, but also to the so-called “pleiotropic” effects, including the influence on subclinical chronic inflammation and haemostasis [2–6].

In particular, statins exert anti-inflammatory properties [7] and inhibit that of the proinflammatory cytokines [8]. The anti-inflammatory effect of statins has been attributed to their ability to reduce, by inhibiting HMG-CoA reductase, not only cholesterol synthesis but also the activation of the mevalonate pathway, with the consequent reduction of isoprenylated and geranylgeranylated proteins, and in particular of Ras prenylation. The statin-induced Ras inhibition reduces the activity of the transcription factor nuclear factor kappa B (NF-kB), which is involved in a wide range of inflammatory pathways and in the formation of reactive oxygen species (ROS) [9]. This statement is confirmed by the observation that high dose simvastatin reduces the binding activity of the proinflammatory transcription factor NF-kB and the concentrations of inflammatory molecules, while the combination of low dose simvastatin with ezetimibe, resulting in a similar low-density lipoprotein (LDL)-cholesterol reduction, does not affect the inflammatory markers [10].

Effects of statins on endothelial function, immunomodulation, and thrombogenesis could play a role in their ability to prevent cardiovascular events, to ameliorate the prognosis of patients affected by acute myocardial infarction, and to reduce the risk of restenosis after coronary angioplasty [11]. In particular, simvastatin has been shown to exert endothelial beneficial effects by promoting nitric oxide production [12], vasorelaxation [13, 14], and improving leukocyte/endothelium interactions [15]. However, it is still debated whether these effects contribute to an additional cardiovascular risk reduction beyond that expected from LDL-cholesterol lowering [11, 16–18].

Links between chronic inflammation and atherothrombosis are very tight [19] and involve platelets, which interact with endothelial cells and leukocytes by adhesion molecules and trigger inflammation by releasing proinflammatory molecules [20–22].

The mechanisms of inflammation-induced thrombosis, including the relationships between inflammation and platelet function, have been reviewed [23].

Platelets derived from patients with elevated plasma LDL-cholesterol show in vitro hyperaggregability, increased fibrinogen binding and surface expression of P-selectin, and increased production of thromboxane A_2_ (TXA_2_) and of superoxide anion, whereas plasma derived from the same patients contains increased concentrations of platelet activation markers, such as soluble CD40 ligand (sCD-40L) and beta-thromboglobulin [24].

The statin-induced reduction of platelet activity has been associated with changes of LDL-cholesterol, oxidized-LDL, and P-selectin [25].

The relationships between statin effects on inflammatory molecules and platelet function have been addressed by very few studies. A peculiar aspect of the antiplatelet effect of statins is their ability to reduce the so-called “aspirin resistance”, a phenomenon associated with adverse cardiovascular outcomes and increased mortality, more frequent in hyperlipidaemic patients than in general population [26]. From the biochemical point of view, elevated cholesterol reduces the aspirin-mediated platelet acetylation, a mechanism involved in the aspirin-induced antiaggregating effect, exerted via the irreversible inhibition of TXA_2_ biosynthesis [27]. Therapy with statins is able to significantly reduce platelet TXA_2_ formation in patients taking low dose of aspirin [28].

Furthermore, in vitro experiments carried out by platelet incubation with pravastatin or simvastatin and aspirin demonstrate that statins improve the aspirin-induced platelet inhibition, suggesting that they directly interact with platelet membranes (though these interactions do not include statin's effects on membrane cholesterol or membrane permeability) and modulate signalling pathways in platelets [29].

On the other hand, some “in vivo” studies demonstrating a platelet inhibiting effect of statins after only few days of treatment suggest an effect largely independent of cholesterol lowering [25, 30, 31]. Thus, some mechanisms involved in platelet inhibition occur before any reduction of cholesterol concentrations.

In summary, literature data show that statins exert a platelet antiaggregating effect, but it is still not clear whether it is mediated by statin effects on LDL-cholesterol, or inflammation, or both, or by direct statin effects on platelets.

We designed this study to investigate the association between the platelet inhibitory effect of simvastatin with the lipid lowering and a wide spectrum of pro- and anti-inflammatory, pro- and anti-atherogenic markers induced by the drug in patients affected by primary hypercholesterolemia.

## 2. Methods

### 2.1. Subjects, Materials, and Methods

We investigated forty-five patients affected by newly diagnosed primary hypercholesterolemia. They did not have a family history of diabetes mellitus and were otherwise healthy on the basis of medical history, physical examination, and standard diagnostic procedures; in particular, they did not present arterial hypertension, impaired fasting glucose, or impaired glucose tolerance measured by the oral glucose tolerance test (OGTT), congestive heart failure, previous peripheral or coronary or cerebral ischemic vascular diseases, endocrine diseases (including hypothyroidism), renal, hepatic, or hepatobiliary diseases, and myopathic or haemostatic disorders. From the study, we also excluded patients on treatment with nonsteroidal anti-inflammatory or antiplatelet drugs, or antioxidant supplements in the previous three weeks. Patients were randomized to be treated with diet plus simvastatin 40 mg/die for two months (n=25) or diet alone without pharmacological intervention (n=20). All patients followed a low-fat diet close to the Adult Treatment Panel (ATP) III guidelines (7% energy from saturated fat and 200 mg dietary cholesterol per day). The study was approved by the Ethics Committee of San Luigi Gonzaga Hospital and all participants authorized data use for investigational purpose by signed informed consent. At baseline and after two months, all subjects were submitted to a clinical evaluation and in fasting venous blood samples we assessed the following parameters.

*(A) Metabolic Parameters and Insulin*. Glucose, total and HDL-cholesterol, triglycerides, and apolipoprotein B (Apo B)-100 were measured by automated chemistry by the Central Laboratory of our hospital. LDL-cholesterol was calculated according to the Friedwald's formula. Insulin was measured by a radioimmunoassay kit (Biochem Immuno System, Bologna, Italy). Insulin sensitivity in the fasting state was estimated using HOMA-IR index by the following formula: fasting plasma glucose (mmol/l) x fasting serum insulin (*μ*U/ml) divided by 22.5. HOMA-IR is commonly used in clinical studies as a reliable marker of insulin resistance [32].

*(B) Markers of Inflammation, Endothelial and Platelet Activation, and Oxidative Stress*. They are as follows: (i) proinflammatory, proatherogenic cytokines and chemokines: Interleukin-1*β* (IL-1*β*), Interleukin-5 (IL-5), Interleukin-6 (IL-6), Interleukin-7 (IL-7), Interleukin-8 (IL-8), Interleukin-9 (IL-9), Interleukin-12 (IL-12), Interleukin-13 (IL-13), Interferon-Inducible Protein (IP-10), Interferon-*γ* (IFN-*γ*), Eotaxin, and Monocyte Chemoattractant Protein-1 (MCP-1); (ii) anti-inflammatory and antiatherogenic markers: Interleukin-10 (IL-10) and Soluble Receptor of Advanced Glycation End Products (sRAGE); (iii) markers of endothelial activation: Vascular Endothelial Growth Factor (VEGF) and soluble E-selectin (sE-selectin); (iv) markers of in vivo platelet activation: soluble P-selectin (sP-selectin), sCD-40L, Platelet Derived Growth Factor-BB (PDGF-BB), and RANTES; (v) a marker of in vivo oxidative stress: 8-hydroxy-2'-deoxyguanosine (8-OHdG).

Serum and plasma samples for biomarkers detection were stored at -80°C until assayed.

Serum concentrations of IL-1*β*, IL-5, IL-6, IL-7, IL-8, IL-9, IL-10, IL-12, IL-13 IFN-*γ*, IP-10, Eotaxin, MCP-1, VEGF, RANTES, and PDGF-bb were measured in duplicate by using the Bio-Plex cytokine assay (Bio-Rad Laboratories Inc., Hercules, CA, USA) according to manufacturer's instructions. The Bio-Plex system combines the principle of a sandwich immunoassay with the Luminex fluorescent-bead-based technology allowing the simultaneous measurement of many cytokines.

Serum levels of sE-selectin, sP-selectin, sRAGE, and sCD40L were measured in duplicate with enzyme-linked immunosorbent assay kits (R&D Systems, Abingdon, United Kingdom) according to manufacturer's instructions.

Serum levels of 8-OH-dG were measured by a competitive enzyme-linked immunosorbent assay (Bioxytech 8-OHdG-EIA, OXIS Health Products, Portland, Oregon).

*(C) Platelet Function Assays*. (i) Platelet aggregation: venous blood samples were withdrawn without stasis and anticoagulated with 3.8% sodium citrate, pH 7.4 (1ml for 9 ml of blood). Platelet-rich plasma (PRP) was obtained by using the Platelet Function Centrifuge (BioData Corporation, Horsham, PA), designed to provide a rapid separation of PRP by a centrifugation for 30 sec. From the top, only two-thirds of the supernatant were collected to avoid contamination by other circulating cells and the remaining blood was further centrifugated for 180 sec to obtain platelet-poor plasma (PPP).

PRP samples were stimulated by arachidonic acid (AA) (1mmol/l), ADP (10 *μ*mol/l), and collagen (4 mg/l) (Mascia Brunelli, Milan, Italy) and platelet aggregation was measured as light-scattering changes by using an eight-channel aggregation system (Platelet Aggregation Profiler, PAP-8, BioData Corporation) according to the Born's method [33]. Platelet aggregation in response to agonists was reported as maximal aggregation (MA). Each aggregation test was recorded for 5 min after the addition of the agonist.

(ii) Platelet sensitivity to aspirin: PRP obtained as described above was stimulated by AA (1 mmol/l) in the absence (see above) and in the presence of a 30 min preincubation with lysine acetylsalicylate (L-ASA) (1-50 *μ*mol/l) (Sanofi-Aventis, Milan, Italy). Platelet aggregation was measured as described above.

### 2.2. Statistical Analysis

Data are expressed as mean ± SD. Normality of data was checked using Shapiro–Wilk test. Continuous data was examined using parametric analyses performed by Student's t-test for paired and unpaired data. Data are given as mean ± standard deviation (SD). Data with a non-Gaussian distribution were analysed using the Mann–Whitney U test and Wilcoxon signed-rank test, as appropriate. Univariate linear regression analysis was performed to assess the correlation of lipid parameters with circulating biomarkers and platelet aggregation. Pearson's correlation was used to examine the significance of correlation between variables. To evaluate the combined effects of different variables on the platelet parameters, we used a multivariate linear regression model with a backward approach. All analyses were performed with SPSS v.24.

## 3. Results

The clinical characteristics of the investigated subjects at baseline and after two months with or without simvastatin therapy are shown in Table 1. At baseline, anthropometric and clinical and metabolic parameters did not significantly differ between the two groups. After two months, in the group of patients treated with simvastatin we found, as expected, a significant reduction of total and LDL-cholesterol and of Apo B-100, whereas no differences were found in control group.

### 3.1. Changes in Inflammatory, Atherogenic, and Oxidative Stress Markers

Table 2 shows circulating levels of inflammatory, atherogenic, and oxidative stress markers before and after the two months of follow-up. At baseline no differences were found between the two groups for each investigated biomarker. After two months, in simvastatin-assigned group, significant reductions of IL-6 (27%, p<0.01), IL-8 (14%, p<0.05), IL-13 (38%, p<0.0001), IFN-*γ* (52%, p<0.0001), and 8-OH-dG (28%, p<0.0001) and significant increases of IL-10 (104%, p<0.0001) and sRAGE (55%, p<0.0001) were found. In control group, all these parameters did not change.

### 3.2. Changes in Endothelial and Platelet Activation Markers

**Figure 1:**
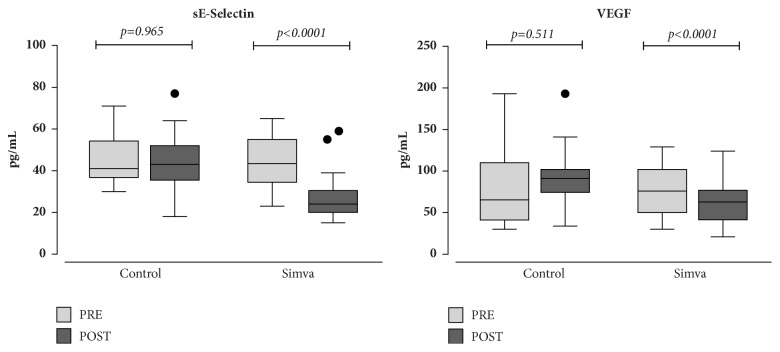
Box-plot analysis of the endothelial dysfunction markers E-selectin and vascular endothelial growth factor (VEGF) in hypercholesterolemic patients assigned to diet alone (Control) or to diet plus simvastatin (Simva) at baseline (pre) and after a two-month follow-up (post). Significance of intragroup differences was estimated by paired t-test or Wilcoxon test, as appropriate. Solid lines: median values; boxes: interquartile range; whiskers: nonoutlier range; closed circles: outliers.

**Figure 2:**
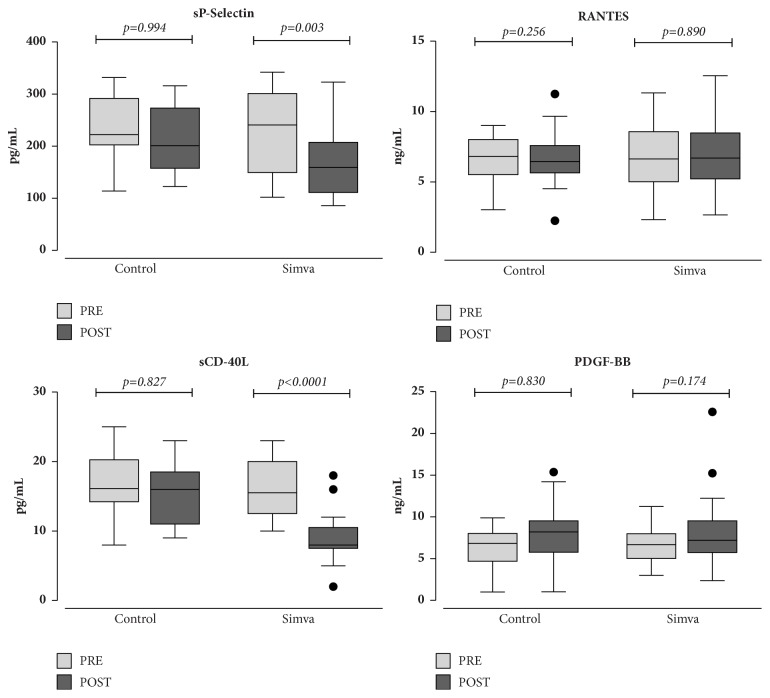
Box-plot analysis of the platelet activation markers soluble P-selectin (sP-selectin), Rantes, soluble CD-40 ligand (sCD-40L), and platelet-derived growth factor (PDGF)-BB, in hypercholesterolemic patients assigned to diet alone (Control) or to diet plus simvastatin (Simva) at baseline (pre) and after a two-month follow-up (post). Significance of intragroup differences was estimated by paired t-test or Wilcoxon test, as appropriate. Solid lines: median values; boxes: interquartile range; whiskers: nonoutlier range; closed circles: outliers.

A two-month treatment with simvastatin induced a significant decrease of the endothelial dysfunction markers sE-selectin (33%, p<0.0001) and VEGF (30%, p<0.0001) (Figure 1). As far as platelet activation markers are concerned, we observed a significant reduction of sP-selectin (21%, p=0.003) and sCD-40L (41%, p<0.0001) whereas RANTES and PDGF-BB did not change (Figure 2). In patients who served as controls, no difference for each investigated parameter was found.

### 3.3. Changes in Platelet Aggregability

**Figure 3:**
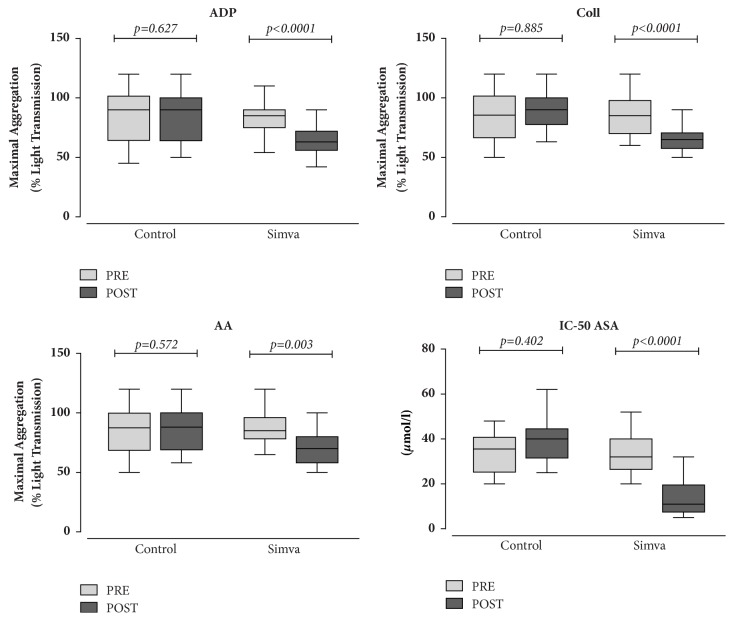
Box-plot analysis showing maximal aggregation to ADP, collagen, arachidonic acid (AA), and lysine acetylsalicylate (L-ASA) half-maximal inhibitory concentration (IC-50) in the presence of AA in hypercholesterolemic patients assigned to diet alone (Control) or to diet plus simvastatin (Simva) at baseline (pre) and after a two-month follow-up (post). Significance of intragroup differences was estimated by paired t-test or Wilcoxon test, as appropriate. Solid lines: median values; boxes: interquartile range; whiskers: nonoutlier range.

As shown in Figure 3, when platelet aggregation tests were evaluated, a two-month treatment with simvastatin resulted in a decrease of MA values in response to ADP (22%, p<0.0001), collagen (21%, p<0.0001), and AA (19%, p<0.0001). In the same subjects, an improvement of the platelet sensitivity to the antiaggregating effects of aspirin was also observed as mirrored by the decrease of L-ASA IC-50 in the presence of AA (65%, p=0.0001). In patients who served as controls, no difference for each investigated platelet function parameter was found.

### 3.4. Correlation Studies

As shown in Table 3, univariate regression analysis between lipid profile and all circulating markers and aggregability parameters of all patients at baseline revealed that LDL-cholesterol levels are (i) positively associated with IFN-*γ*, IL-6, VEGF, E-selectin, and sCD-40L as circulating markers of inflammation, endothelial dysfunction, and* in vivo* platelet activation, respectively, 8-OH-dG as marker of oxidative stress, platelet aggregation to ADP, collagen, AA, and IC-50 L-ASA and (ii) inversely associated with the anti-inflammatory markers IL-10 and sRAGE. No significant correlations were found for triglycerides levels (data not shown). Interestingly, when a similar analysis was carried out on the data after simvastatin we found a positive correlation between LDL-cholesterol and E-selectin (r=0.616, p=0.001) and 8-OH-dG (r=0.500, p=0.011).

Correlation between platelet aggregability and the investigated pattern of circulating biomarkers in all patients at baseline is shown in Table 4. Of note, independently of proaggregating stimulus, an increased platelet aggregation and a reduced antiaggregating effect of aspirin were associated with increase of E-selectin, IFN-*γ*, and IL-6, thus pointing out the relationships between impaired endothelial function, inflammation, and platelet hyperaggregability. Interestingly, in this context also reduced levels of the anti-inflammatory sRAGE played a role in increasing platelet response to activators (ADP, AA) and decreasing the inhibitory effect of L-ASA.

When the differences between values at baseline and after two months of simvastatin treatment (delta values) of LDL-cholesterol were correlated with the delta values of all the evaluated parameters, no significant correlation was found (data not shown).

Table 5 shows the multiple linear regression analysis with MA to ADP, collagen, and AA and with L-ASA IC-50 to AA entered as dependent variables and the parameters significantly correlated with them (see Table 4) entered as independent variables.

The significant predictors were for MA to ADP, LDL-cholesterol, VEGF, p-selectin, sRAGE; for MA to collagen, LDL-cholesterol alone; for MA to AA, LDL-cholesterol, IL-1b, IL-10, and sCD40L; and for L-ASA IC-50 to AA, LDL-cholesterol and sCD40L.

## 4. Discussion

This study, carried out in patients with primary hypercholesterolemia, shows that a two-month therapy with simvastatin improved not only, as expected, the lipid profile but also a wide pattern of proatherogenic and prothrombotic parameters. Actually, simvastatin treatment reduced mediators of inflammation, oxidative stress, and endothelial and platelet activation, increased anti-inflammatory circulating markers, reduced platelet aggregating responses to ADP, collagen, and AA and increased platelet sensitivity to aspirin.

To the best of our knowledge, this is the first study which simultaneously considered a so wide spectrum of the potential simvastatin effects, trying to correlate them with the platelet effects of the drug.

On the other hand, at baseline, a correlation was observed between LDL-cholesterol levels and markers of inflammation (such as IL-6 and IFN-g), of anti-inflammation (such as IL-10 and sRAGE), endothelial activation markers (such as sE-selectin, VEGF), platelet activation markers (such as sCD-40L), oxidative stress (such as 8-OH-dG), and platelet function parameters evaluated in terms of aggregation to activators (i.e., collagen, AA, and ADP) or response to inhibitor (i.e., L-ASA). The biological explanation of this phenomenon is attributable to the role exerted by LDL-cholesterol on subclinical inflammation, oxidative stress, and platelet activation, as previously mentioned.

The role of LDL-cholesterol on platelets is suggested by evidence showing that lipoprotein disorders affect platelet function. Actually, LDL particles sensitize platelets by the binding of apoB-100 to the specific receptor on the platelet membrane and the subsequent modification of platelet function via a wide spectrum of interactions: in particular, LDL particles in their native form induce hypersensitivity of platelets to agonists resulting in increased aggregation and secretion responses whereas, after oxidation, they become independent platelet activators in stirred platelet suspensions [34].

In our study, when platelet function parameters were correlated with biomarkers at baseline, we observed the occurrence of significant correlations not only with LDL-cholesterol, but also with proinflammatory (such as IL-1b, IL-6, and IFN-*γ*), anti-inflammatory (such as IL-10 and sRAGE), endothelial activation (such as sE-selectin, VEGF), and platelet activation (sP-selectin, sCD-40L, and PDGF-BB) markers. In the multivariate analysis, LDL-cholesterol was the parameter that more strongly influenced platelet sensitivity to activators (ADP, collagen, and AA) and inhibitor (aspirin) suggesting a primary role for LDL-cholesterol in determining the cascade of inflammatory events responsible also for the activated platelet function.

In our study, lack of correlations between most of biomarkers levels and LDL-cholesterol after simvastatin suggests that some pleiotropic effects of simvastatin may be independent of their effects on LDL-cholesterol levels. However, a positive and significant correlation after simvastatin was found with E-selectin and 8-OH-dG and this fact induces hypothesizing that the improvement of cholesterol levels may alone influence some peculiar aspects of endothelial function and oxidative stress.

On the other hand, we did not observe significant correlations between delta values of LDL- cholesterol and delta values of all the parameters modified by the simvastatin treatment (data not shown). Although this fact is not surprising and fits with other observations in literature, we are aware, however, that the limited number of subjects enrolled in our study could be a possible explanation for the absence of significant correlations between delta values of LDL- cholesterol and delta values of all the parameters modified after statin treatment. However, from the biological point of view, the plausibility of this phenomenon could be also due to the fact that the mechanisms by which statins exert the lipid-lowering and the pleiotropic effects are different, although the first step is the inhibition of HMG-CoA reductase. Furthermore, the demonstration of a direct effect of the statins on platelets observed in “in vitro” experiments [29, 35] further increases the complexity of the picture.

Of course, the interplay between inflammation and thrombosis is enhanced in the presence of dyslipidemia: not surprisingly, statins, which reduce both lipid concentrations and inflammation, are considered antithrombotic drugs, as reviewed [36, 37].

The so-called “pleiotropic effects” of statins on endothelial function, vascular inflammation, immunomodulation, and thrombogenesis could play a role in their ability to prevent cardiovascular events, to ameliorate the prognosis of patients affected by acute myocardial infarction, and to reduce the risk of restenosis after angioplasty, as reviewed [17], even if it is still debated whether these effects contribute an additional cardiovascular risk reduction beyond that expected from LDL-cholesterol lowering [11, 16–18].

A previous study demonstrated that simvastatin inhibits TXA_2_ biosynthesis and platelet function in hypercholesterolemic patients [38, 39]. Also other statins reduce platelet function. In particular, fluvastatin therapy reduced platelet cholesterol/phospholipid molar ratio and platelet aggregation, suggesting that its antiaggregating effect is due to reduced platelet cholesterol content [40]. Atorvastatin therapy reduced collagen-induced platelet aggregation [41], platelet activation evaluated by flow cytometry [42], and platelet function in patients with coronary heart disease [43]. Pravastatin, as well as simvastatin, reduced platelet thrombus formation after only 8 weeks of therapy [44]. The statin-induced reduction of platelet activity has been associated with changes of LDL-cholesterol, oxidized-LDL, and P-selectin [25]. Our study extends previous studies by showing the inhibiting effects of simvastatin treatment on pathways of platelet aggregation activated by three different agonists and on the L-ASA antiaggregating effect. Furthermore, we observed that simvastatin reduced sCD40L and sP-selectin levels as markers of platelet activation.

As previously mentioned, statins are able to reduce the so-called “aspirin resistance”, a phenomenon associated with adverse cardiovascular outcomes and increased mortality [26]. Actually, in our study simvastatin therapy showed the ability to significantly improve the in vitro platelet sensitivity to the antiaggregating effects of aspirin, a finding consistent with data obtained by other authors showing a reduced TXA_2_ formation in patients treated with simvastatin and receiving aspirin before and after statin administration [28].

In the present study we observed an effect of simvastatin treatment on a variety of markers of inflammation and we evaluated the relationships between the statin effects on inflammatory molecules and platelet function, an aspect addressed by few studies. It has been shown that eight weeks of treatment with atorvastatin and rosuvastatin are associated with comparable reductions in LDL-cholesterol, high sensitive C reactive protein (hsCRP), 11-dehydro-TXB_2_ (a marker of TXA_2_ biosynthesis), and 8-iso-prostaglandin F_2*α*_ (a marker of lipid peroxidation with platelet-activating properties): in this study, in multiple regression analyses, only hsCRP and LDL-cholesterol were independent predictors of 11-dehydro-TXB_2_, while only LDL-cholesterol predicted 8-iso-PGF_2*α*_ [45]. Notably, we showed for the first time that simvastatin treatment is also able to significantly reduce circulating sRAGE concentrations. It is known that sRAGE levels are reduced in hypercholesterolemic patients as compared to healthy subjects and inversely correlate with urinary excretion of isoprostanes and plasma asymmetric dimethyl-arginine suggesting that the ligand-RAGE axis may link endothelial dysfunction with oxidative stress [46].

## 5. Conclusions

In conclusion, this study provides the first evidence that a short-term treatment with simvastatin simultaneously affects a wide range of markers of inflammation and atherothrombosis, adding a piece of information to better clarify the rationale of simvastatin therapy in patients at a high cardiovascular risk. In fact, simvastatin therapy, beside its hypocholesterolemic effect, (i) decreases oxidative stress, proatherogenic and proinflammatory markers, (ii) increases antiatherogenic and anti-inflammatory markers, (iii) reduces platelet aggregation to physiological agonists, and (iv) increases platelet sensitivity to the antiaggregating effects of aspirin. In this scenario, LDL-cholesterol levels are a major correlate and possibly a determinant of enhanced platelet reactivity suggesting a primary role for LDL-cholesterol in determining the cascade of inflammatory events responsible also for the impaired platelet function.

## Figures and Tables

**Table 1:** Clinical characteristics of hypercholesterolemic patients at baseline and after two months of treatment with diet alone (control) or simvastatin.

	**Control (n=20)**			**Simvastatin (n=25)**		
	**Before**	**After**	**p-value**	**Before**	**After**	**p-value**
**Male/Female**	M=9 / F=11	-	-	M=11 / F=14	-	-
**Age (years)**	55±11	-	-	59±13	-	-
**BMI (kg/m** ^**2**^ **)**	25±3	25±3	0.4913	25±4	25±4	0.3698
**TC (mg/dl)**	270±43	269±50	0.8300	282±30	185±27	<0.0001
**HDL-C (mg/dl)**	47±5	48±4	0.1523	48±9	48±7	0.3851
**LDL-C-C (mg/dl)**	188±45	185±51	0.7584	196±33	107±25	<0.0001
**APO B-100 (mg/dl)**	157±17	155±16	0.3153	152±17	93±15	<0.0001
**TG (mg/dl)**	177±44	175±48	0.7146	190±61	149±43	0.0027
**FG (mg/dl)**	87±9	85±8	0.3372	88±9	88±8	0.8580
**HOMA-IR**	2.6±1.3	2.5±1	0.4331	2.6±1.2	2.4±1	0.9678
**SBP (mm Hg)**	123±7	124±7	0.7199	126±12	125±10	0.2075
**DBP(mmHg)**	81±6	80±4	0.7239	79±7	79±7	0.9715

Data are presented as mean±SD. TC, total cholesterol; HDL-C, high-density lipoprotein cholesterol; LDL-C, low-density lipoprotein cholesterol; APO B, apolipoprotein B; TG, triglycerides; FG; fasting glucose; SBP, systolic blood pressure; DBP, diastolic blood pressure. For p value, paired Student's t-test or Wilcoxon test was used as appropriate.

**Table 2:** Pro- and anti-inflammatory and oxidative stress markers in subjects at baseline and after two months of treatment with diet alone (control) or simvastatin.

	**Control (n=20)**			**Simvastatin (n=25)**		
	**Before**	**After**	**p value**	**Before**	**After**	**p value**
**Pro-inflammatory**						
**IL-1b **	0.98±0.63	0.94±0.46	0.5008	0.86±0.45	0.79±0.41	0.3625
**IL-5**	1.8±0.7	1.6±0.7	0.1043	2.0±1.7	2.2±1.4	0.1742
**IL-6**	6.7±2.5	6.7±2.4	0.6404	6.6±3.4	4.8±2.2	0.0034
**IL-7**	5.9±2.1	5.7±1.0	0.6676	5.6±2.5	4.9±1.9	0.2143
**IL-8**	16.8±4.3	15.6±4.0	0.1919	18.9±6.0	16.3±6.7	0.0447
**IL-9**	18.5±6.6	17.8±7.6	0.4428	17.7±11.6	19.0±15.2	0.6186
**IL-12**	22.8±7.3	21.4±6.8	0.5124	22.0±13	22.8±13	0.7145
**IL-13**	5.9±2.2	6.0±2.8	0.6864	7.3±4.3	4.5±2.7	<0.0001
**IFN-** **γ**	32.7±14.4	34.0±16.2	0.6744	38.1±13.6	18.3±7.5	<0.0001
**IP-10**	519±226	476±195	0.1960	491±219	521±264	0.6189
**Eotaxin **	177±63	177±46	0.7509	143±67	167±54	0.0534
**MCP-1**	57±23	60±16	0.5257	60±45	50±31	0.1906

**Anti-Inflammatory**						
**IL-10**	1.07±0.55	1.04±0.51	0.7046	1.03±0.56	2.10±0.51	<0.0001
**sRAGE**	678±268	659±248	0.4637	740±263	1147±310	<0.0001

**Oxidative Stress**						
**8-OH-dG**	1.60±0.56	1.62±0.58	0.9038	1.84±0.59	1.33±0.56	<0.0001

Data are presented as mean ± SD. IL, interleukin; MCP, Monocyte Chemoattractant Protein; sRAGE, Soluble Receptor of Advanced Glycation End Products; 8-OH-dG, 8-hydroxy-2'-deoxyguanosine. Concentrations are expressed as pg/ml, except where otherwise indicated. For p value, paired Student's t-test or Wilcoxon test was used as appropriate.

**Table 3:** Correlation between LDL-C and pro- and anti-inflammatory, oxidative stress, and platelet activation markers in hypercholesterolemic patients at baseline.

	**LDL-C**	
	**r**	**p**
**IFN-** **γ**	0.493	0.001
**IL-6**	0.427	0.003
**VEGF**	0.301	0.044
**E-Selectin**	0.560	<0.0001
**sCD-40L**	0.348	0.019

**IL-10**	-0.364	0.014
**sRAGE**	-0.484	0.001

**8-OH-dG**	0.307	0.040

**MA ADP**	0.638	<0.0001
**MA COLL**	0.614	<0.0001
**MA AA**	0.623	<0.0001
**IC-50 L-ASA**	0.600	<0.0001

IFN, interferon; IL, interleukin; VEGF, vascular endothelial growth factor; sCD-40L, soluble CD-40 ligand; IL, interleukin; sRAGE, Soluble Receptor of Advanced Glycation End Products; 8-OH-dG, 8-hydroxy-2'-deoxyguanosine; MA, maximal aggregation; Coll, collagen; AA, arachidonic acid; IC-50, half-maximal inhibitory concentration; L-ASA, lysine acetylsalicylate.

**Table:** (a) MA to ADP

	**r**	**p**
**IFN-** **γ**	0.391	0.008
**IL-6**	0.384	0.009
**VEGF**	0.326	0.029
**P-Selectin**	0.326	0.029
**E-Selectin**	0.432	0.003
**sRAGE**	-0.453	0.002

**Table:** (b) MA to collagen

	**r**	**p**
**IFN-** **γ**	0.326	0.029
**IL-6**	0.327	0.028
**PDGF-BB**	0.331	0.027
**E-Selectin**	0.419	0.004

**Table:** (c) MA to AA

	**r**	**p**
**IFN-** **γ**	0.315	0.035
**IL-10**	-0.448	0.002
**IL-1b**	0.320	0.032
**IL-6**	0.425	0.004
**E-Selectin**	0.515	0.0003
**sCD-40L**	0.562	<0.0001
**sRAGE**	-0.515	0.0003

**Table:** (d) L-ASA IC-50 to AA

	**r**	**p**
**IL-6**	0.347	0.020
**E-Selectin**	0.339	0.023
**sCD-40L**	0.442	0.002
**sRAGE**	-0.454	0.002

TC, total cholesterol; LDL-C, low-density lipoprotein; IFN, interferon; IL, interleukin; VEGF, vascular endothelial growth factor; sCD-40L, soluble CD-40 ligand; IL, interleukin; sRAGE, Soluble Receptor of Advanced Glycation End Products; PDGF, Platelet Derived Growth Factor; Coll, collagen; AA, arachidonic acid; IC-50, half-maximal inhibitory concentration; L-ASA, lysine acetylsalicylate.

**Table:** (a) MA to ADP

MA to ADP	Adjusted R^2^=0.362	F=25.954	p<0.0001
	Constant	B=7.072	p=0.7299
	LDL-C	B=0.250	p=0.0016
	VEGF	B=0.191	p=0.0081
	p-Selectin	B=0.134	p=0.0009
	sRAGE	B=-0.021	p=0.0510

**Table:** (b) MA to collagen

MA to Collagen	Adjusted R^2^=0.393	F=15.239	p<0.0001
	Constant	B=31.722	p=0.0069
	LDL-C	B=0.290	p<0.0001

**Table:** (c) MA to AA

MA to AA	Adjusted R^2^=0.615	F=18.580	p<0.0001
	Constant	B=21.873	p=0.0946
	LDL-C	B=0.211	p=0.0003
	IL-10	B=-8.762	p=0.0240
	IL-1b	B=9.012	p=0.0128
	sCD-40L	B=1.549	p=0.0013

**Table:** (d) L-ASA IC-50 to AA

L-ASA IC-50 to AA	Adjusted R^2^=0.394	F=15.302	p<0.0001
	Constant	B=1.406	p=0.8282
	LDL-C	B=0.132	p=0.0002
	sCD-40L	B=0.609	p=0.0398

## Data Availability

The data used to support the findings of this study are available from the corresponding author upon request.
